# The Angiogenic Potential of DPSCs and SCAPs in an *In Vivo* Model of Dental Pulp Regeneration

**DOI:** 10.1155/2017/2582080

**Published:** 2017-09-05

**Authors:** Petra Hilkens, Annelies Bronckaers, Jessica Ratajczak, Pascal Gervois, Esther Wolfs, Ivo Lambrichts

**Affiliations:** Laboratory of Morphology, Biomedical Research Institute (BIOMED), Hasselt University, Diepenbeek, Belgium

## Abstract

Adequate vascularization, a restricting factor for the survival of engineered tissues, is often promoted by the addition of stem cells or the appropriate angiogenic growth factors. In this study, human dental pulp stem cells (DPSCs) and stem cells from the apical papilla (SCAPs) were applied in an *in vivo* model of dental pulp regeneration in order to compare their regenerative potential and confirm their previously demonstrated paracrine angiogenic properties. 3D-printed hydroxyapatite scaffolds containing DPSCs and/or SCAPs were subcutaneously transplanted into immunocompromised mice. After twelve weeks, histological and ultrastructural analysis demonstrated the regeneration of vascularized pulp-like tissue as well as mineralized tissue formation in all stem cell constructs. Despite the secretion of vascular endothelial growth factor *in vitro*, the stem cell constructs did not display a higher vascularization rate in comparison to control conditions. Similar results were found after eight weeks, which suggests both osteogenic/odontogenic differentiation of the transplanted stem cells and the promotion of angiogenesis in this particular setting. In conclusion, this is the first study to demonstrate the successful formation of vascularized pulp-like tissue in 3D-printed scaffolds containing dental stem cells, emphasizing the promising role of this approach in dental tissue engineering.

## 1. Introduction

Sufficient vascular supply is a restricting factor for the survival of engineered and transplanted tissues. Within the field of regenerative dentistry and the treatment of necrotic, immature, permanent teeth in particular, the establishment of adequate tissue vascularization remains a challenge [1]. The currently applied regenerative endodontic procedures (REP) comprise the induction of a blood clot, causing both the release of sequestered growth factors and the attraction of endogenous (stem) cells, such as stem cells from the apical papilla (SCAPs) [2, 3]. However, in addition to the questionable histological nature of the regenerated tissues [4, 5], adequate pulp tissue revascularization is an often recurring concern in cell homing-based approaches given the limited vascular access through the apical foramen [6].

Although a number of studies suggest a positive correlation between pulp revascularization and an increasing size of the root apex [6–8], the use of stem cell-based approaches has demonstrated the successful regeneration of vascularized dental pulp-like tissue in canine teeth with a small apical opening. Transplantation of dental pulp stem cells (DPSCs) even led to a more distinct volume of regenerated dental tissue with a higher capillary density in comparison to a growth factor-based approach [9]. These data are in accordance with the previously demonstrated angiogenic properties of dental stem cells (DSCs) *in vitro* and *in vivo*. Recent work from our group and others reported that both DPSCs and SCAPs express a wide range of regulatory angiogenic proteins, including but not limited to vascular endothelial growth factor (VEGF), basic fibroblast growth factor (bFGF), angiopoietin-1, matrix metalloproteinases, endostatin, thrombospondin-1, and insulin-like growth factor-binding protein-3 [10–17]. In addition to the secretion of the aforementioned factors, numerous studies have reported the paracrine influence of DPSCs and SCAPs on endothelial proliferation, migration, and tube formation both *in vitro* and *in vivo*, for example, in a rat model of myocardial infarction [10, 12, 14, 18–22].

With regard to the application of (dental) stem cells in dental pulp regeneration, the ectopic root transplantation model, that is, the transplantation of emptied human root canals, is considered to be a widely applied proof-of-principle model [1]. Notwithstanding the elaborate application of this animal model as well as the fact that it takes into consideration the limited dental vascular supply, the size and shape of human root canals are very prone to variability [19, 23–25]. Three-dimensional (3D) printing or additive manufacturing, that is, the inkjet-based production of 3D structures by printing liquid binders onto loose powders, provides an alternative means to create custom-made, inorganic scaffolds with a controllable chemistry and porosity. As 3D printing allows the use of a wide variety of biomaterials, such as bioactive glasses, polylactic acid (PLA), polyethylene glycol (PEG), or hydroxyapatite (HA), it has a great clinical potential in the field of organ and tissue replacement [26, 27]. Mannoor et al., for example, reconstructed a bionic ear through 3D printing of a cell-seeded hydrogel matrix [28]. Khalyfa and coworkers reported the production of craniofacial segments based on calcium phosphate powders [29]. Given the broad availability of biomaterials as well as the ability to produce scaffolds with a custom-made design according to the patient's needs, 3D printing also shows great promise in the field of regenerative dentistry [30, 31]. Chiu et al., for example, recently reported the successful osteogenic differentiation of DPSCs in cultured 3D-printed mineral trioxide aggregate/polycaprolactone scaffolds [32]. Similar results were found by Louvrier and coworkers, indicating the *in vitro* odontoblastic differentiation potential of DPSCs in a 3D-printed polycaprolactone-based scaffold [33]. With regard to the *in vivo* potential of 3D-printed scaffolds, Wei et al. described the formation of a bio-root complex after in situ transplantation of HA/tricalcium phosphate scaffolds containing DPSCs and periodontal ligament stem cells in pigs [34]. Furthermore, recent studies also demonstrated the creation of vascular templates, facilitating the perfusion of tissue constructs and thus ameliorating cell viability [35, 36].

This study aims to investigate the regenerative potential of DPSCs and SCAPs and their ability to sustain their paracrine angiogenic potential in an ectopic transplantation model of dental pulp regeneration. Since the biocompatibility and effectiveness of HA has already been demonstrated *in vivo* [34, 37–39], 3D-printed, conically shaped HA scaffolds in order to mimic the tooth's morphology and to minimize scaffold-related variability. To our knowledge, this is one of the first studies that combines DSCs with the aforementioned scaffolds in a model for dental pulp tissue engineering. DPSCs and SCAPs are supported by a commercially available, self-assembling peptide hydrogel, which is proven to maintain DSC viability both *in vitro* and *in vivo* [19, 24, 40]. Constructs are characterized by both scanning electron microscopy and secretion of VEGF. After twelve and eight weeks of transplantation into immunocompromised mice, tissue content and vascularization are assessed at the histological and ultrastructural level. Given the previously demonstrated angiogenic properties of DPSCs and SCAPs as well as their potential to regenerate dental tissues, we expect to observe the successful regeneration of a strongly vascularized dentin/pulp-like complex after transplantation of 3D-printed HA scaffolds containing DSCs.

## 2. Materials and Methods

### 2.1. Cell Culture

Dental tissues were acquired with informed consent from patients (15–20 years of age, male and female) undergoing extraction of third molars for therapeutic or orthodontic reasons at Ziekenhuis Maas en Kempen, Bree and Ziekenhuis Oost-Limburg, Genk, Belgium. Written informed consent of underaged patients was obtained through their guardians, as approved by the medical ethical committee of Hasselt University, Belgium (protocol 13/0104U). The apical papilla was immediately resected from the tooth after extraction, while the pulp tissue was obtained with forceps after mechanically fracturing the disinfected tooth with surgical chisels. Dental tissues were rinsed and transported at 37°C in Minimal Essential Medium Eagle, alpha modification (*α*MEM, Sigma-Aldrich, St. Louis, USA) supplemented with 2 mM L-glutamine (Sigma-Aldrich), 100 U/ml penicillin, 100 *μ*g/ml streptomycin (Sigma-Aldrich), and 10% fetal bovine serum (FBS, Gibco, ThermoFisher Scientific, Waltham, USA). This medium is further referred to as standard DSC culture medium. DPSCs and SCAPs were isolated according to the explant method as described previously by our group [41]. Briefly, dental tissues were cut into 1-2 mm^3^ fragments, which were cultured in standard DSC culture medium for 10–14 days to allow cellular outgrowth. When 80% confluency was reached, DSCs were retrieved from the culture plates by means of 0.05% trypsin/EDTA and subcultured for further experiments. All DSC cultures were tested for the expression of the following (stem) cell markers at protein level by means of flow cytometry as described previously [41]: CD31, CD34, CD45, CD73, CD90, and CD105. In order to minimize patient-related variability and the amount of animals required for the ectopic transplantation model, DPSCs and SCAPs (passage 1) of three different donors were pooled and maintained in standard DSC culture medium. The pooled stem cells were cryopreserved in liquid nitrogen for further experiments. All experiments were performed with thawed passage 2-3 DPSCs and SCAPs.

### 2.2. Scaffold Production and Characterization

**Figure 1:**
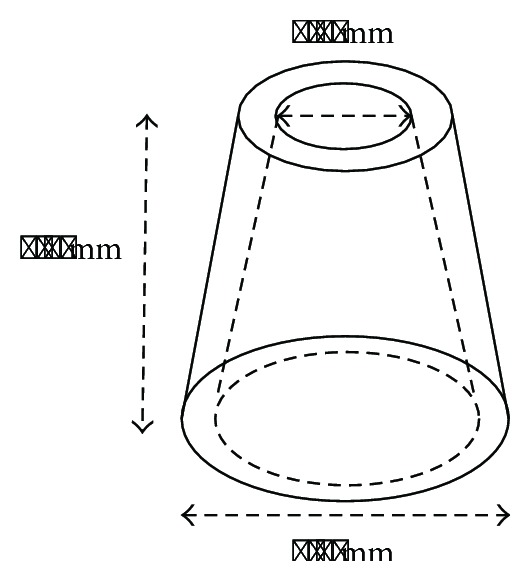
Dimensions of 3D-printed, conically shaped HA scaffolds. Height: 4.9 mm; base diameter: 4.3 mm; and opening diameter: 1.7 mm.

In order to mimic the size and shape of a tooth root, 3D-printed, conically shaped HA constructs with a standardized length of 4.9 mm and a representative apical opening of 1.7 mm were used in this study (Figure 1) [6–8]. All scaffolds were custom-made by Sirris (Seraing, Belgium). Briefly, constructs were created in a layer-by-layer fashion through additive manufacturing using a paste containing hydroxyapatite/tricalcium phosphate (HA/TCP) powder, resins, and an ultraviolet (UV) photoinitiator. Polymerization of each layer was achieved by means of UV light, after which the scaffolds were cleaned. All scaffolds were thermally treated in order to remove potential residues of organic material. Finally, the scaffolds were sintered to increase their density. Scanning electron microscopy (SEM) was performed to assess the surface characteristics of the constructs.

### 2.3. Assessment of Vascular Endothelial Growth Factor Secretion

In order to evaluate the survival and secretion of VEGF by DSCs within the scaffolds and the supporting hydrogel, an enzyme-linked immunosorbent assay (ELISA) against VEGF (Raybiotech, Norcross, GA, USA) was performed on conditioned medium of constructs containing 50,000 DPSCs according to the manufacturer's instructions. Briefly, scaffolds were injected with a 1 : 1 solution of phosphate-buffered saline (PBS) and pooled DPSCs in 0.2% Puramatrix™ peptide hydrogel (Corning Life Sciences, Tewksbury, MA, USA), after which they were maintained in standard DSC culture medium during four weeks. After 14 days and 28 days of culturing, conditioned medium was obtained by incubating the constructs for 48 h with standard DSC culture medium containing 0.1% FBS.

### 2.4. Ectopic Transplantation Model

Scaffolds containing either 0.2% hydrogel (Puramatrix, negative control condition) or a 0.2% hydrogel suspension combined with 25,000 DPSCs and 25,000 SCAPs or a 1 : 1 mixture of DPSCs and SCAPs were subcutaneously transplanted into the dorsum of 8-week-old, female, severe combined immunodeficient (SCID) hairless outbred (SHO™) mice (SHO-Prkdc^scid^Hr^hr^, Charles River, Wilmington, MA, USA). Following eight (*n* = 24, six animals per group) or twelve (*n* = 32, eight animals per group) weeks of transplantation, the constructs were resected from the mice and processed for histological stainings and ultrastructural analysis as described below. Before sectioning, macroscopic signs of blood vessel growth were assessed by means of a stereomicroscope (Wild M3Z Stereomicroscope, Heerbrugg, Switzerland) equipped with a Nikon digital net camera DN100 (Nikon Co., Shinjuku, Tokyo, Japan). This study was approved by the Ethical Committee on Animal Experiments (ECAE) of Hasselt University, Belgium (protocol 201332V1).

### 2.5. Histological Stainings

Resected constructs (*n* = 20, eight weeks, and *n* = 24, twelve weeks) were fixed with 4% paraformaldehyde (PFA, Sigma-Aldrich, St. Louis, USA) for 48 h at room temperature. Following fixation, the base of the conically shaped scaffolds was removed with a surgical saw, to allow complete impregnation of the formed tissue in paraffin. The resulting cylinders were dehydrated in graded alcohol and embedded in paraffin (Paraplast Plus, Surgipath, Leica Biosystems, Wetzlar, Germany). Paraffin-embedded constructs were decalcified (decalcifying solution, Thermo Scientific™, Richard-Allan Scientific, Waltham, MA, USA) for 24 h at room temperature, after which the paraffin was melted again to allow better impregnation of the decalcified constructs. Afterwards, a hematoxylin and eosin (H&E) staining as well as a Masson's trichrome staining was performed on serial tissue sections (7 *μ*m). Sections were scanned with a Mirax digital slide scanner (Carl Zeiss Vision, Aalen, Germany), and the amount of blood vessels was determined for each tissue section by counting the blood vessels in the entire construct by means of Panoramic Viewer software (3DHISTECH Ltd., Budapest, Hungary). In order to determine the percentage of newly formed tissue, the ratio of the measured surface of newly formed tissue (mm^2^) and the total inner surface area (mm^2^) of each tissue section was calculated. The vascularization rate was defined by the ratio of the amount of blood vessels in each tissue section and the measured surface of newly formed tissue (mm^2^).

### 2.6. Ultrastructural Analysis

Resected constructs (*n* = 4, eight weeks and *n* = 8, twelve weeks) were fixed with 2% glutaraldehyde in 0.05 M sodium cacodylate buffer (pH 7.3) at 4°C. Following fixation, the base of the conical scaffolds was removed with a surgical saw, to allow better postfixation. The resulting cylinders were treated with 2% osmium tetroxide in 0.05 M sodium cacodylate buffer (pH 7.3) for 1 h at 4°C. The constructs were stained with 2% uranyl acetate in 10% acetone for 20 min, after which they were dehydrated with ascending concentrations of acetone. The dehydrated samples were impregnated overnight in 1 : 1 mixture of acetone and araldite epoxy resin at room temperature. Following impregnation, the samples were embedded in araldite epoxy resin at 60°C. Araldite-embedded constructs were decalcified (decalcifying solution, Thermo Scientific) for 24 h, after which they were refilled with araldite and cut into semithin (0.5 *μ*m) and thin sections (70 nm) with a Leica EM UC6 microtome. Semithin sections were stained with thionine methylene blue, and representative pictures were taken with a Nikon Eclipse 80i microscope equipped with a DS-5 M digital camera. Thin sections were transferred to 0.7% formvar-coated copper grids (Aurion, Wageningen, The Netherlands) and contrasted with 0.5% uranyl acetate and a stabilized solution of lead citrate by means of a Leica EM AC20. Transmission electron microscopy (TEM) was performed with a Philips EM208 S electron microscope (Philips, Eindhoven, The Netherlands). Representative digital images were obtained with a Morada Soft Imaging System camera and the corresponding ITEM-FEI software (Olympus SIS, Münster, Germany).

### 2.7. Exclusion Criteria and Statistics

Scaffolds that were compromised during tissue processing were excluded from the study (*n* = 5, twelve weeks). Constructs containing less than 10% newly formed tissue were considered to be empty and excluded from further characterization and parameter calculation (*n* = 3, eight and twelve weeks). Statistical analysis was performed using GraphPad Prism 5 Software (GraphPad Software, La Jolla, CA, USA). Data distribution was assessed by means of a D'Agostino & Pearson normality test. As *n* < 8 in each group, data distribution was considered to be nonparametric. Data were compared with a Kruskall-Wallis test combined with a Dunn's posthoc test. Differences were considered to be statistically significant at *P* values ≤ 0.05. All data were expressed as mean ± standard deviation (SD). ^∗^*P* value < 0.05; ^∗∗^*P* value < 0.01; and ^∗∗∗^*P* value < 0.001.

## 3. Results

### 3.1. Characterization of Dental Stem Cells

**Figure 2:**
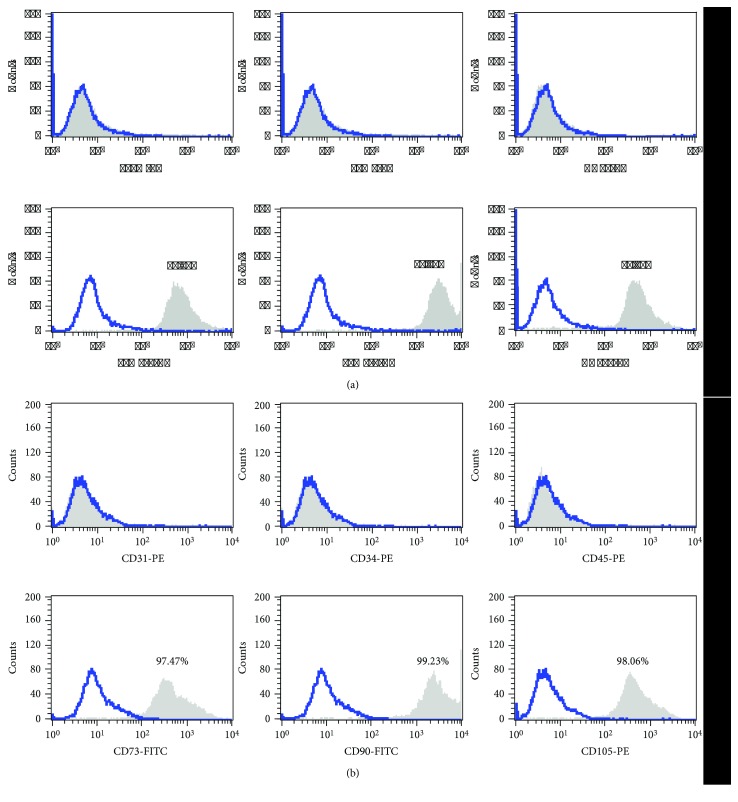
Expression of stem cell markers by dental stem cells. (a) Dental pulp stem cells. (b) Stem cells from the apical papilla. Both dental stem cell populations showed a pronounced expression of CD73, CD90, and CD105 and were negative for CD31, CD34, and CD45. Matched isotype controls are shown in blue.

Flow cytometry analysis indicated the expression of typical stem cell markers by DSCs (Figure 2). Both DPSCs (Figure 2(a)) and SCAPs (Figure 2(b)) showed a pronounced expression of CD73, CD90, and CD105, while they were negative for CD31, CD34, and CD45.

### 3.2. Characterization of Stem Cell Constructs

**Figure 3:**
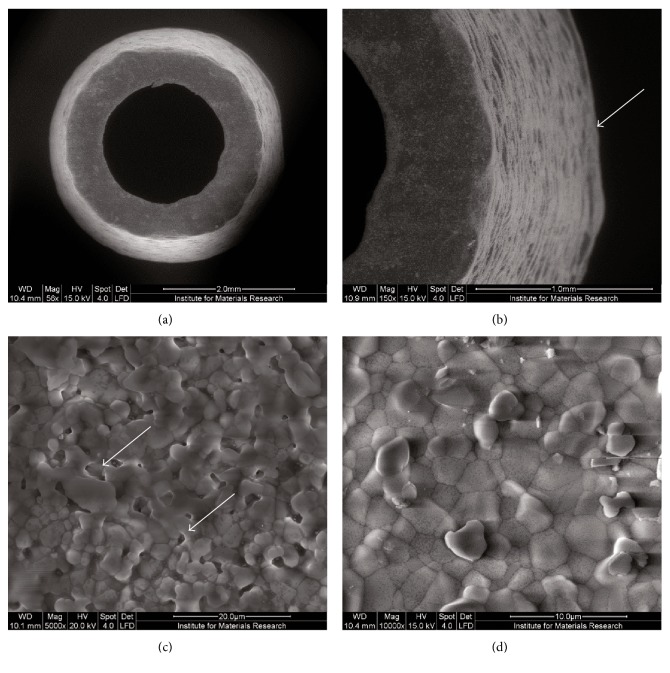
Surface characterization of 3D-printed hydroxyapatite (HA) constructs. (a) Overview. Scale bar = 2.0 mm. (b) Distinct layers of deposited HA (arrow). Scale bar = 1.0 mm. (c) Pseudoporous surface (arrows). Scale bar = 20 *μ*m. (d) Dense HA layer without clearly distinguishable pores. Scale bar = 10 *μ*m.

SEM analysis was performed in order to obtain a detailed view of both the scaffolds' structure and surface characteristics (Figure 3). In addition to distinct layers of deposited HA (arrow, Figure 3(b)), a pseudoporous surface was also visible (Figure 3(c)). However, in-depth analysis of the constructs indicated rather dense HA layers, without clearly distinguishable pores (Figure 3(d)).

**Figure 4:**
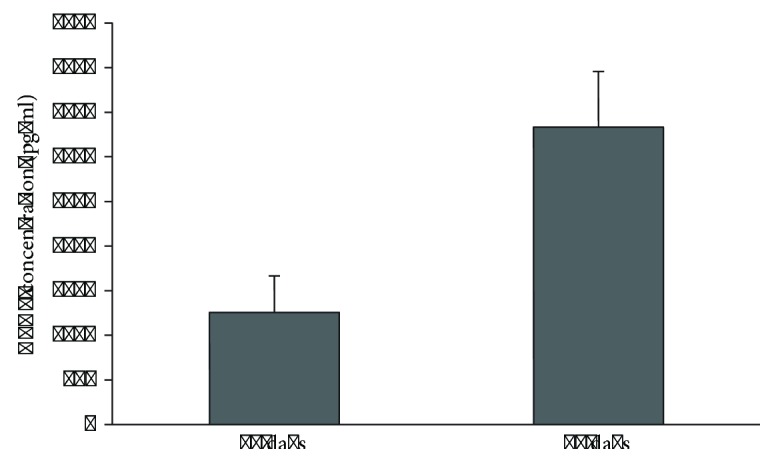
VEGF secretion of dental pulp stem cells in HA constructs. DPSCs resuspended in 0.2% peptide hydrogel contained within 3D-printed HA scaffolds showed an increasing concentration of VEGF detected within the conditioned medium after 14 and 28 days of culturing. This assay was performed on two replicate scaffolds containing pooled DPSCs. Data are represented as mean ± standard deviation.

The angiogenic properties of DPSCs within the scaffolds were evaluated by means of an ELISA against VEGF. After 14 days of culturing, the conditioned medium of the constructs contained a substantial amount of VEGF. VEGF secretion increased further after 28 days of culturing (Figure 4).

### 3.3. Evaluation of Vascularized Tissue Regeneration after Twelve Weeks of Transplantation

**Figure 5:**
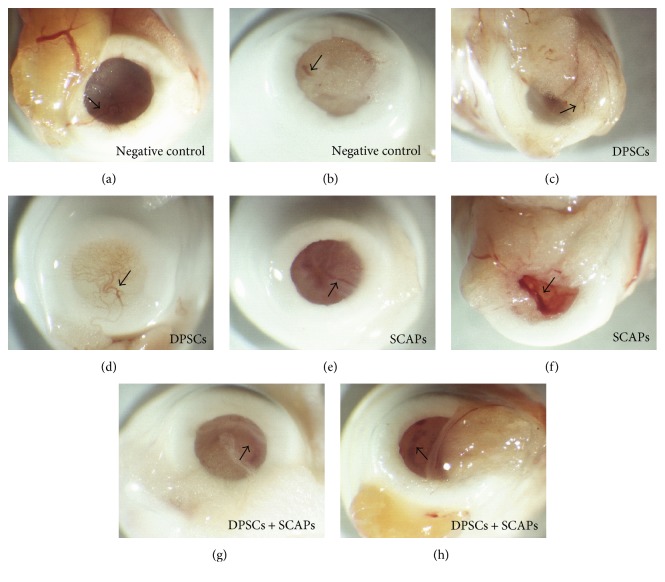
Macroscopic assessment of blood vessel ingrowth after 12 weeks of transplantation. (a, b) Negative control condition (Puramatrix). (c, d) DPSCs. (e, f) SCAPs. (g, h) DPSCs + SCAPs. Approximately all the resected constructs showed clear signs of blood vessel ingrowth (arrows). Experimental groups as well as samples within the same experimental condition showed notable differences in terms of tissue color and the mount of visible blood vessels. Representative pictures were taken of eight constructs/experimental group.

After 12 weeks of transplantation, all constructs were resected and assessed for macroscopic signs of blood vessel ingrowth (Figure 5). Although blood vessel ingrowth was present in approximately all the resected constructs, the color of the tissue as well as the distinct presence of blood vessels differed not only between the experimental groups (negative control condition (Figures 5(a) and 5(b)); DPSCs (Figures 5(c) and 5(d)); SCAPs (Figures 5(e) and 5(f)); and DPSCs + SCAPs (Figures 5(g) and 5(h))) but also within the samples of the same experimental condition.

**Figure 6:**
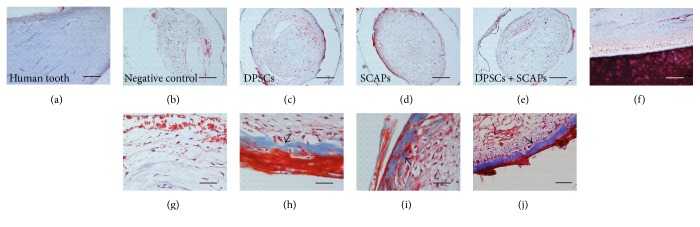
Histological characterization of 3D constructs. (a, f) Human tooth. (b, g) Negative control condition (Puramatrix). (c, h) DPSCs. (d, i) SCAPs. (e, j) DPSCs + SCAPs. In all stem cell constructs (c–e), vascularized pulp-like tissue was formed with a notably lower collagen content in comparison to human dental pulp tissue (a). Negative control constructs also contained vascularized fibrous tissue, albeit with a different organizational structure (b, g). Strongly organized, concentric layers of collagen and mineralized tissue were found in constructs containing stem cells (h–j, arrows). Scale bar = 500 *μ*m (b–e); 200 *μ*m (a, f); 100 *μ*m (j); and 50 *μ*m (g–i). Analysis was conducted on scaffolds with at least 10% newly formed tissue. Representative pictures were taken of four constructs/experimental group.

With regard to the histological characterization of the 3D constructs, Masson's trichrome staining indicated the formation of a vascularized pulp-like tissue in all the constructs containing stem cells (Figures 6(c), 6(d), and 6(e)). Although the newly formed tissue was structurally similar to human dental pulp tissue (Figures 6(a) and 6(f)), it contained notably less collagen. The negative control constructs (0.2% Puramatrix without cells), on the other hand, also comprised vascularized, fibrous tissue, albeit with a different organizational structure (Figures 6(b) and 6(g)). While the outer tissue border of the stem cell constructs showed strongly organized, concentric layers of collagen and, in certain cases, mineralized tissue formation (Figures 6(h), 6(i), and 6(j), arrows), only two out of four negative control constructs showed loosely arranged, concentric layers of collagen (Figure 6(g)).

**Figure 7:**
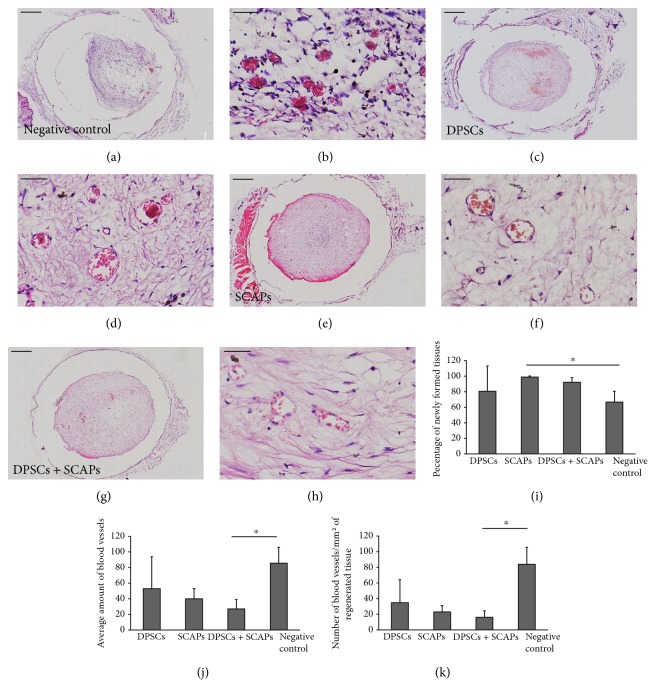
Tissue and blood vessel content of 3D constructs. (a, b) Negative control condition (Puramatrix). (c, d) DPSCs. (e, f) SCAPs. (g, h) DPSCs + SCAPs. All experimental conditions contained blood vessels of variable shapes and sizes. Scale bar = 500 *μ*m (a, c, e, g); 50 *μ*m (b, d, f, h). Representative pictures were taken of four constructs/experimental group. (i) Percentage of newly formed tissue within the 3D constructs. Negative control constructs contained significantly less tissue in comparison to constructs containing SCAPs. (j) Number of blood vessels with the 3D constructs. Negative control constructs contained a significantly higher amount of blood vessels in comparison to constructs containing DPSCs + SCAPs. (k) Number of blood vessels per mm^2^ of newly formed tissue. The vascularization rate of the negative control constructs was significantly higher in comparison to that of the constructs containing DPSCs + SCAPs. Analysis was conducted on four constructs/experimental group, with each sample containing more than 10% newly formed tissue. ^∗^*P* value < 0.05.

Microscopical analysis of H&E-stained tissue sections (Figure 7(i)) demonstrated a significantly lower percentage of newly formed tissue within the negative control condition (0.2% Puramatrix without cells) in comparison to the scaffolds containing stem cells, in particular SCAPs. In terms of blood vessel content, the presence of small as well as larger (sometimes ruptured) blood vessels was demonstrated in all experimental conditions (Figures 7(a), 7(b), 7(c), 7(d), 7(e), 7(f), 7(g), and 7(h)). While containing a significantly lower amount of tissue (Figure 7(i)), the negative control condition in particular seemed to contain a significantly higher amount of blood vessels (Figure 7(j)). Similar observations were made when calculating the amount of blood vessels per mm^2^ of newly formed tissue (Figure 7(k)).

### 3.4. Ultrastructural Analysis of 3D Constructs after Twelve Weeks of Transplantation

**Figure 8:**
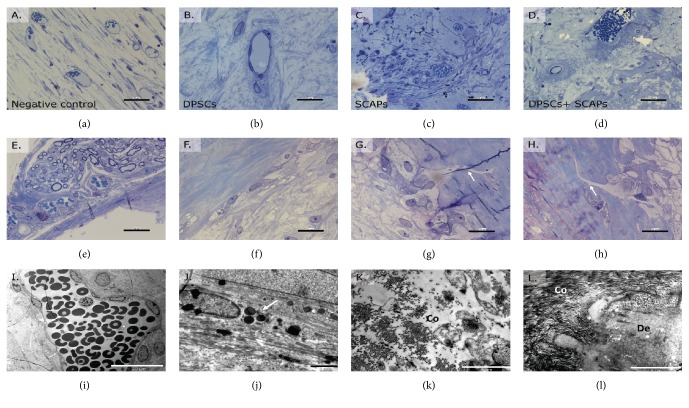
Ultrastructural analysis of 3D constructs after 12 weeks of transplantation. (a) Negative control condition (Puramatrix). (b) DPSCs. (c) SCAPs. (d) DPSCs + SCAPs. All constructs contained differentially organized, vascularized connective tissue. (e) Nerve fibers were observed in the tissue surrounding the constructs. (f–h) Mineralized tissue was clearly present in DPSC constructs and sometimes contained cellular protrusions (white arrows). (i) Mature blood vessels of variable shapes and size were found in all experimental conditions. (j) Samples contained metabolically active cells, some of which comprised electron-dense vesicles (white arrow). (k) Active collagen secretion was also present in all constructs (Co). (l) Deposits of collagen, hydroxyapatite, and mineralized tissue were only present in the samples containing stem cells (De). Scale bar = 50 *μ*m (a–d); 20 *μ*m (e–i); 5 *μ*m (l); 2 *μ*m (j-k). Analysis was performed on two samples of each experimental condition.

Microscopical analysis of thionine methylene blue-stained semithin tissue sections confirmed the aforementioned histological characterization of the 3D constructs, indicating the presence of differentially organized connective tissue in all of the samples (Figures 8(a), 8(b), 8(c), and 8(d)). Distinct patches of mineralized tissue were present in constructs containing DPSC + SCAPs (Figure 8(d)) and DPSCs (Figures 8(f), 8(g), and 8(h)), respectively. The cells within the constructs were mostly aligned against the mineralized tissue (Figure 8(f)), although in certain regions cellular protrusions were visible within the tissue (Figures 8(g) and 8(h), arrow). Nerve fibers were also detected in the tissue surrounding the outer rim of all the constructs (Figure 8(e)). At the ultrastructural level, mature blood vessels of variable shapes and sizes were observed in all vesicles (Figure 8(j), arrow); all samples showed signs of active collagen secretion (Figures 8(k) and 8(l), Co). Tissue deposits containing HA needles and collagen in various stages of mineralization were clearly present in the constructs containing stem cells (Figure 8(l), De). This was not the case in the negative control sample (0.2% Puramatrix without cells), which only contained patches of collagen secretion (Figure 8(k)).

Both the significantly lower amount of blood vessels and the lower vascularization rate in the stem cell constructs in comparison to those of the negative control condition after twelve weeks of transplantation (Figure 7) suggest that the time frame of transplantation might play a role in the attraction of endogenous cells and/or vascular supply in the negative control constructs. Since angiogenesis is a self-limiting biological process, the angiogenic potential of the stem cells may have reached a plateau phase during these twelve weeks and thereby stimulated the inherent hard tissue-forming capacity of DPSCs and SCAPs, as demonstrated by the patches of mineralized tissue formation in all stem cell constructs (Figure 8). Therefore, the experimental set-up was repeated with similar conditions for a shortened period of time, that is, eight weeks.

### 3.5. Histological and Ultrastructural Characterization of 3D Constructs after Eight Weeks of Transplantation

**Figure 9:**
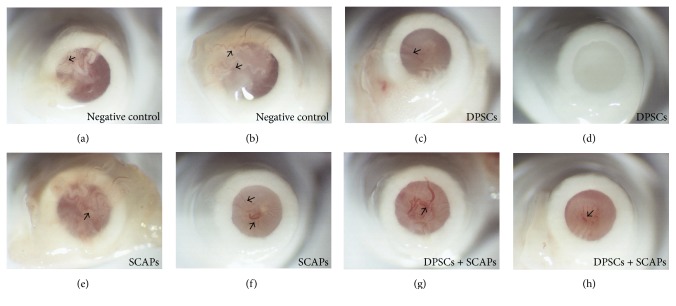
Macroscopic assessment of blood vessel ingrowth after eight weeks of transplantation. (a, b) Negative control condition (Puramatrix). (c, d) DPSCs. (e, f) SCAPs. (g, h) DPSCs + SCAPs. Approximately all the resected constructs showed clear signs of blood vessel ingrowth (arrows). Experimental groups as well as samples within the same experimental condition showed notable differences in terms of tissue color and the mount of visible blood vessels. Representative pictures were taken of six constructs/experimental group.

After eight weeks of transplantation, all constructs were resected and assessed for macroscopic signs of bloods vessel ingrowth (Figure 9, arrows). Similar to the 12-week transplantation period, blood vessel in-growth could be observed in most of the resected constructs, with notable differences between experimental groups and within constructs of the same experimental condition with regard to the color of the tissue and the amount of visible blood vessels.

**Figure 10:**
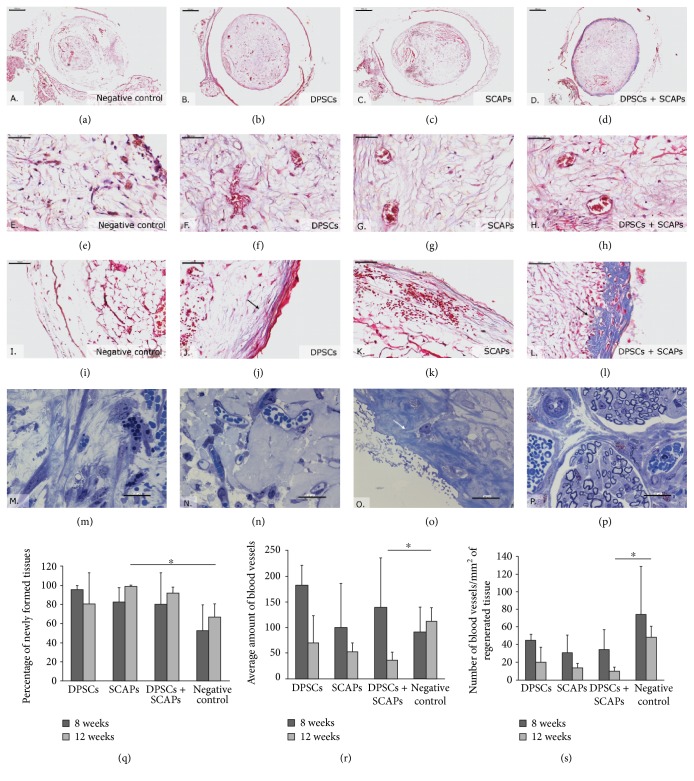
Tissue and blood vessel content of 3D constructs after eight weeks of transplantation. (a, e, i) Negative control condition (Puramatrix). (b, f, j) DPSCs. (c, g, k) SCAPs. (d, h, l) DPSCs + SCAPs. (a–h) All constructs contained differentially organized, vascularized connective tissue. (j–o) Strongly organized, concentric layers of collagen or mineralized tissue (arrows) were found in constructs containing stem cells. (p) Nerves were observed in the tissue surrounding the constructs. Scale bar = 500 *μ*m (a–d); 100 *μ*m (i); 50 *μ*m (e–h, j–l); and 20 *μ*m (m–p). Representative pictures were taken of each experimental condition. (q) Percentage of newly formed tissue within 3D constructs. Negative control constructs seemed to contain less tissue in comparison to the stem cell constructs. (r) Number of blood vessels with the 3D constructs. DPSC constructs showed a trend towards a higher amount of blood vessels in comparison to the negative control constructs. (s) Number of blood vessels per mm^2^ of newly formed tissue. The vascularization rate of the negative control constructs appeared higher in comparison to that of the stem cell constructs. Analysis after eight weeks was conducted on *n* = 3 (DPSCs); *n* = 5 (SCAPs); *n* = 5 (DPSCs + SCAPs); and *n* = 4 (negative control condition), with each sample containing more than 10% newly formed tissue. ^∗^*P* value < 0.05.

In terms of histological characterization, both the H&E-stained and Masson's trichrome-stained tissue sections were similar to the ones of the 12-week transplantation period (Figures 10(a), 10(b), 10(c), 10(d), 10(e), 10(f), 10(g), 10(h), 10(i), 10(j), 10(k), and 10(l)). While the stem cell constructs contained vascularized pulp-like tissue structurally similar to human dental pulp tissue (Figures 10(b), 10(c), 10(d), 10(f), 10(g), and 10(h)), negative control constructs (0.2% Puramatrix without cells) comprised vascularized, fibrous tissue with a notably different structural organization (Figures 10(a) and 10(e)). The outer border of the stem cell constructs showed either strongly organized, concentric layers of collagen (SCAPs, Figure 10(k)) or mineralized tissue formation (DPSCs or DPSCs + SCAPs, Figures 10(j) and 10(l), arrows). Negative control constructs comprised very loosely arranged collagen fibers (Figure 10(i)), with the exception of one sample which seemed to contain substantial amount of calcified tissue (data not shown). With regard to the percentage of newly formed tissue within the constructs, microscopical analysis demonstrated a trend similar to the 12-week transplantation period, although no statistically significant differences were detected between the experimental conditions (Figures 10(a), 10(b), 10(c), 10(d), and 10(q)). In contrast to the 12-week transplantation period, analysis of the blood vessel content showed a seemingly, but not significantly, higher amount of blood vessels in the constructs containing DPSCs (Figure 10(r)). However, calculation of the vascularization rate demonstrated a trend towards higher amount of blood vessels per mm^2^ of newly formed tissue in the negative control condition, albeit with a considerable variability between the different samples (Figure 10(s)).

Regarding the ultrastructural analysis, thionine methylene blue-stained semithin tissue sections pointed out that only two (DPSCs and DPSCs + SCAPs) out of four processed samples showed signs of new tissue formation. Either active collagen secretion or distinct patches of mineralized tissue were present in the vascularized tissue (Figures 10(m) and 10(n)) or mineralized tissue with cellular protrusions (Figure 10(o), arrow). Nerve fibers were only detected in the tissue surrounding the outer border of two out of four samples (Figure 10(p)).

## 4. Discussion

Within the field of regenerative endodontic procedures and dental tissue engineering, the limited vascular supply of the tooth due to the size of the root apex is an often recurring concern, in particular in the treatment of necrotic, immature permanent teeth [1, 6, 10]. However, Takeuchi and coworkers reported the successful regeneration of highly vascularized dental pulp tissue after transplantation of DPSCs in root canals with an apical opening of 0.7 mm [9]. Our group previously demonstrated the angiogenic properties of DPSCs and SCAPs *in vitro* as well as in an *in vivo*-like setting [10]. In order to investigate whether these stem cells sustain their angiogenic potential in a standardized, *in vivo* setting, an ectopic transplantation model of dental pulp regeneration was performed in immunocompromised mice. More specifically, conically shaped, 3D-printed scaffolds containing a self-assembling peptide hydrogel (Puramatrix) and/or DPSCs, SCAPs, or DPSCs + SCAPs were subcutaneously transplanted in SCID mice for eight or twelve weeks.

After a transplantation period of twelve weeks, histological analysis demonstrated the formation of vascularized pulp-like tissue in all constructs containing stem cells. Although structurally similar to human dental pulp tissue, the newly formed tissue displayed a notably lower collagen content. Similar findings were reported previously by Rosa et al. and others, indicating a more densely organized extracellular matrix in human dental pulps [24, 25]. This enhanced matrix formation was also observed after transplantation of human root canals comprising cocultures of DPSCs and endothelial cells [19]. Despite being contained within multiple layers of hydroxyapatite, DPSCs were still able to secrete substantial amounts of VEGF *in vitro*, as demonstrated by ELISA. *In vivo*, however, stem cell constructs did not display a larger amount of blood vessels in comparison to the negative control condition after twelve weeks of transplantation. While the latter comprised on average 20% less tissue, the average number of blood vessels per mm^2^ of newly formed tissue was significantly higher, in particular in comparison to that of the condition containing DPSCs + SCAPs. In contrast, Dissanayaka et al. reported the complete absence of vascularized pulp-like tissue in their control, that is, sealed human root canals containing Puramatrix, after four weeks of transplantation [19]. Rosa and coworkers, on the other hand, observed a minimal amount of poorly organized tissue in open human root canals containing the same self-assembling peptide hydrogel devoid of stem cells after 35 days of transplantation [24].

As mentioned earlier, the applied 12-week transplantation period in this study might have played a role in the attraction of endogenous cells and the vascularization of the negative control constructs. Furthermore, due to the relatively long time period, the angiogenic potential of DPSCs and SCAPs could have stagnated and caused the stimulation of the inherent odontogenic and/or osteogenic differentiation capacity of the stem cells, as demonstrated by the distinct regions of mineralized tissue formation within the stem cell constructs. Therefore, an intermediate transplantation period of eight weeks was chosen to repeat the experimental set-up.

Histological and ultrastructural characterization of these constructs, however, confirmed the presence of vascularized pulp-like tissue with patches of mineralized tissue formation in the stem cell constructs, similar to the tissue formed after twelve weeks of transplantation. Although DPSC constructs did contain a seemingly higher amount of blood vessels, a trend towards a lower vascularization rate within the stem cell constructs was observed after calculation of the number of blood vessels per mm^2^ of newly formed tissue. Next to the determining role of the transplantation period, these results suggest that the intrinsic properties of the scaffold itself could have influenced the behavior of the transplanted cells substantially. Hydroxyapatite, for example, displays a distinct osteoinductive capacity and has been shown to promote the osteogenic differentiation of mesenchymal stem cells [42, 43]. The absence of soluble dentin matrix components, on the other hand, might explain the lower vascularization rate of the stem cell constructs as these proteins are expected to have additional proangiogenic effects together with the transplanted DPSCs and SCAPs [44]. Angiogenic growth factors such as VEGF and bFGF can also stimulate the hard tissue-forming capacity of mesenchymal stem cells [45–48]. Lentiviral transfection of VEGF, for example, resulted in the successful odontogenic differentiation of human DPSCs *in vitro* [48]. An autoregulatory mechanism reduces the proangiogenic potential of the differentiated cells as stated by Hoch et al., who demonstrated a significant reduction of both VEGF mRNA levels and angiogenesis-promoting effects *in vitro* of osteogenically differentiated mesenchymal stem cells [49]. Another important aspect one has to keep in mind in tissue engineering and transplantation studies is the number of cells related to the size of the scaffold, as a high cell seeding density can be a defining factor for the differentiation potential of mesenchymal stem cells in general [50, 51].

In addition to being strongly vascularized, dental pulp is also a highly innervated tissue. However, after eight as well as twelve weeks of transplantation, nerve fibers were only present in the tissue closely surrounding the constructs. Given the fact that previously published data only concerned newly formed tissue within the transplanted constructs, it is difficult to compare these results. However, the presence and ingrowth of nerve fibers in vascularized pulp-like tissue has been mentioned by Iohara and co-workers, following a two-week transplantation period of a DPSC subpopulation in pulpectomized dog teeth [52, 53]. As these nerve fibers originated from apical tissue, these results indicate that the attraction or regeneration of nerve fibers requires specific cues from the oral environment, which are seemingly absent in the subcutaneous environment of the ectopic root transplantation model.

Taken together, these data confirm the ability of DPSCs and SCAPs to regenerate vascularized pulp-like tissue in 3D-printed HA scaffolds. Although DPSCs and SCAPs were able to sustain their angiogenic potential to a certain degree *in vivo*, the microenvironment at the time of transplantation also promoted the inherent hard tissue-forming potential of the stem cells, which potentially diminished the induction of angiogenesis.

## 5. Conclusion

Despite their previously demonstrated angiogenic potential *in vitro*, constructs containing DPSCs and/or SCAPs did not display a higher vascularization rate in comparison to control constructs, which was probably due to odontogenic and/or osteogenic differentiation of the stem cells. Given its determining role in cellular differentiation and paracrine properties, a stable microenvironment has to be recreated at the time of regeneration. More specifically, in order to create a balance between the maximum angiogenic potential of DPSCS and their inherent hard tissue-forming capacity, the right scaffold has to be combined with the right (amount of) stem cells within the most optimal time frame to establish ideal circumstances for regeneration. Nevertheless, this study shows the successful formation of vascularized dentin/pulp-like tissue after transplantation of 3D-printed hydroxyapatite scaffolds containing DPSCs and/or SCAPs. This approach opens up new perspectives for the application of 3D-printed stem cell constructs in the field of dental tissue engineering.
